# Genetic characterization of a locus responsible for low pungency using EMS-induced mutants in *Capsicum annuum* L.

**DOI:** 10.1007/s00122-024-04602-3

**Published:** 2024-04-12

**Authors:** Seungki Back, Jung-Min Kim, Hayoung Choi, Joung-Ho Lee, Koeun Han, Doyeon Hwang, Jin-Kyung Kwon, Byoung-Cheorl Kang

**Affiliations:** Department of Agriculture, Forestry and Bioresources, Research Institute of Agriculture and Life Science, Plant Genomics and Breeding Institute, College of Agriculture and Life Science, Seoul National University, Seoul, 08826 Republic of Korea

## Abstract

**Key message:**

The pepper mutants (‘221-2-1a’ and ‘1559-1-2h’) with very low pungency were genetically characterized. The* Pun4* locus, responsible for the reduced pungency of the mutant fruits, was localized to a 208 Mb region on chromosome 6. DEMF06G16460, encoding 3-ketoacyl-CoA synthase, was proposed as a strong candidate gene based on the genetic analyses of bulked segregants, DEG, and expression analyses.

**Abstract:**

Capsaicinoids are unique alkaloids present in pepper (*Capsicum* spp.), synthesized through the condensation of by-products from the phenylpropanoid and branched-chain fatty acid pathways, and accumulating in the placenta. In this study, we characterized two allelic ethyl methanesulfonate-induced mutant lines with extremely low pungency (‘221-2-1a’ and ‘1559-1-2h′). These mutants, derived from the pungent Korean landrace 'Yuwolcho,' exhibited lower capsaicinoid content than Yuwolcho but still contained a small amount of capsaicinoid with functional capsaicinoid biosynthetic genes. Genetic crosses between the mutants and Yuwolcho or pungent lines indicated that a single recessive mutation was responsible for the low-pungency phenotype of mutant 221-2-1a; we named the causal locus *Pungency 4* (*Pun4*). To identify *Pun4*, we combined genome-wide polymorphism analysis and transcriptome analysis with bulked-segregant analysis. We narrowed down the location of *Pun4* to a 208-Mb region on chromosome 6 containing five candidate genes, of which *DEMF06G16460*, encoding a 3-ketoacyl-CoA synthase associated with branched-chain fatty acid biosynthesis, is the most likely candidate for *Pun4*. The expression of capsaicinoid biosynthetic genes in placental tissues in Yuwolcho and the mutant was consistent with the branched-chain fatty acid pathway playing a pivotal role in the lower pungency observed in the mutant. We also obtained a list of differentially expressed genes in placental tissues between the mutant and Yuwolcho, from which we selected candidate genes using gene co-expression analysis. In summary, we characterized the capsaicinoid biosynthesis-related locus *Pun4* through integrated of genetic, genomic, and transcriptome analyses. These findings will contribute to our understanding of capsaicinoid biosynthesis in pepper.

**Supplementary Information:**

The online version contains supplementary material available at 10.1007/s00122-024-04602-3.

## Introduction

The *Capsicum* genus includes approximately 42 species and is believed to have originated in tropical and temperate Central and South America, Mexico, and the West Indies of North America (Barboza et al. 2019, 2020; Eshbaugh 1980; Walsh and Hoot 2001). Five *Capsicum* species have been extensively cultivated and domesticated throughout Europe, Africa, India, China, and South America: chili pepper (*C. annuum*), habanero pepper (*C. chinense*), tabasco pepper (*C. frutescens*), cayenne pepper (*C*. *baccatum*), and tree pepper (*C*. *pubescens*) (Barboza et al. 2020; Bosland 1994; Carrizo García et al. 2016). Pepper fruits are used in various ways, ranging from crunchy vegetables to seasonings. The spicy compound used for seasoning in pepper belongs to the capsaicinoids, unique alkaloid mixtures found only in the genus *Capsicum* (Aza-González et al. 2011). Capsaicinoids have numerous functions relevant in human health, including analgesic, anti-cancer, anti-inflammatory, anti-oxidative, and anti-obesity properties (Aza-González et al. 2011; Govindarajan and Sathyanarayana 1991; Liu and Nair 2010; Luo et al. 2011; Negulesco et al. 1987). Furthermore, capsaicinoids exert anti-fungal and anti-oomycete effects, including against *Fusarium* (Veloso et al. 2014). In light of these attributes, extensive research has explored the genetic mechanisms underlying capsaicinoid biosynthesis and regulation.

The existence of the enzyme capsaicin synthase (CS) was proposed based on the chemical structure of capsaicin, as an attempt to explain the condensation reaction leading to the formation of capsaicin (Nelson and Dawson 1923). The capsaicinoid biosynthetic pathway branches in two directions: the phenylpropanoid pathway for vanillylamine biosynthesis and the branched-chain fatty acid pathway for the production of 8-methyl-6-nonenoic acid (Bennett and Kirby 1968; Leete and Louden 1968). The hypothesis of a CS enzyme paved the way for identifying potential capsaicinoid biosynthetic enzymes and their corresponding encoding genes, by harnessing transcriptome and genomic information (Aluru et al. 2003; Curry et al. 1999; Kim et al. 2001). In the phenylpropanoid pathway, well-characterized and conserved precursor enzymes contribute to vanillylamine biosynthesis, including phenylalanine ammonia lyase (PAL), cinnamate 4-hydroxylase (C4H), 4-coumaronyl-CoA ligase (4CL), coumarate 3-hydroxylase (C3H), caffeoyl-CoA *O*-methyltransferase (CCoAOMT), hydroxycinnamoyl-CoA hydratase/lyase (HCHL), and putative aminotransferase (pAMT) (Aza-González et al. 2011; Fujiwake et al. 1982; Mazourek et al. 2009; Stewart Jr et al. 2005; Sukrasno and Yeoman 1993). Similarly, several structural genes have been identified from the branched fatty acid pathway, including those encoding acyl carrier protein (ACL), acyl-ACP thioesterase (FatA), ketoacyl-ACP synthase (KAS), branched-chain amino acid aminotransferase (BCAT), and ketoacyl-ACP reductase (CaKR1) (Aluru et al. 2003; Koeda et al. 2019; Mazourek et al. 2009). Furthermore, genes regulating capsaicinoid biosynthesis, such as transcription factor genes from the *R2R3-MYB* (Han et al. 2019; Liu et al. 2022; Sun et al. 2019; Yu et al. 2023; Zhu et al. 2019) and *WRKY* families (Zhang et al. 2023; Zhu et al. 2019) participate in the regulatory mechanisms behind these two biosynthetic branches. Nonetheless, the functions of only a few genes in the pathway, such as *Pun1* (also known as *C*), *pAMT*, *Pun3* (also known as *CaMYB31*), and *CaKR1*, have been confirmed using natural loss-of-function variants. (Han et al. 2019; Koeda et al. 2019; Lang et al. 2009Stewart Jr et al. 2005). However, due to limitations in available natural variants, it is necessary to utilize artificially induced mutant populations to further elucidate the biosynthetic genes of capsaicinoids.

Several attempts have been made to create mutagenized populations within *Capsicum* species and look for phenotypes of interest, primarily using *C. annuum* cultivars as starting materials. One such early effort involved treating the blocky *C. annuum* cultivar ‘Maor’ with ethyl methanesulfonate (EMS), focusing primarily on mutants with altered shoot architecture (Paran et al. 2007). Subsequently, three different populations have been generated using EMS- or radiation-induced mutations by two separate Korean groups (Hwang et al. 2014; Jo et al. 2016; Siddique et al. 2020). In particular, our group generated EMS-induced populations using the Korean *C. annuum* landrace ‘Yuwolcho’ and the ornamental cultivar ‘Micro-Pep Red.’ We undertook a systematic characterization of phenotypes in this mutant population, including plant growth and development of leaves, flowers, and fruits, in addition to assessing capsaicinoid content (Hwang 2014; Hwang et al. 2014; Jeong et al. 2012b; Siddique et al. 2020). More recently, even reproductive phenotypes, including seed number, have attracted interest (Arisha et al. 2015; Tanaka et al. 2021). To harness the potential of the mutant resources generated through mutagenesis in genetic research, both forward and reverse genetic approaches can be employed. Forward genetics is the main method currently in use to uncover the genetic basis of specific traits. Conversely, reverse genetics is suitable for testing the contribution of a candidate gene to a phenotype of interest and exploring novel allelic variants. High-throughput sequencing techniques have greatly facilitated the identification of causal mutations in species with high-quality genomes, with two main possible approaches (Kim et al. 2014, 2017; Lee et al. 2022; Ou et al. 2018; Qin et al. 2014). For example, the causal mutation behind a phenotype of interest can be rapidly identified by bulked-segregant analysis (BSA) followed by sequencing, named ‘fast-forward genetics,’ which expedites the identification of candidate genes (Schneeberger and Weigel 2011). Through the above BSA-seq approach, a mapping interval can be accurately delimited to a small region while all sequence polymorphisms across the genome can be cataloged, thus enabling the swift identification of candidate genes (Klein et al. 2018).

In our previous studies, we generated an EMS-induced mutant population in the pungent Korean landrace Yuwolcho (Hwang et al. 2014; Jeong et al. 2012a). Among the resulting individuals, we screened 917 M_2_ lines for pungency-related phenotypes. Among the potential mutants, we selected two lines, ‘221-2-1a’ and ‘1559-1-2h,’ with significantly lower capsaicinoid content compared to Yuwolcho, for further investigation (Hwang 2014; Lee 2018). In this study, we performed a comprehensive analysis of the mutant phenotypes and determined the mode of inheritance of these two allelic mutations in different segregating populations. Subsequently, we employed BSA-seq to precisely map the mutant locus and narrow down the candidates. Furthermore, we explored the candidate genes responsible for the low-pungency phenotype of these two mutants using transcriptome analyses.

## Materials and methods

### Plant materials and allelism test

**Fig. 1:**
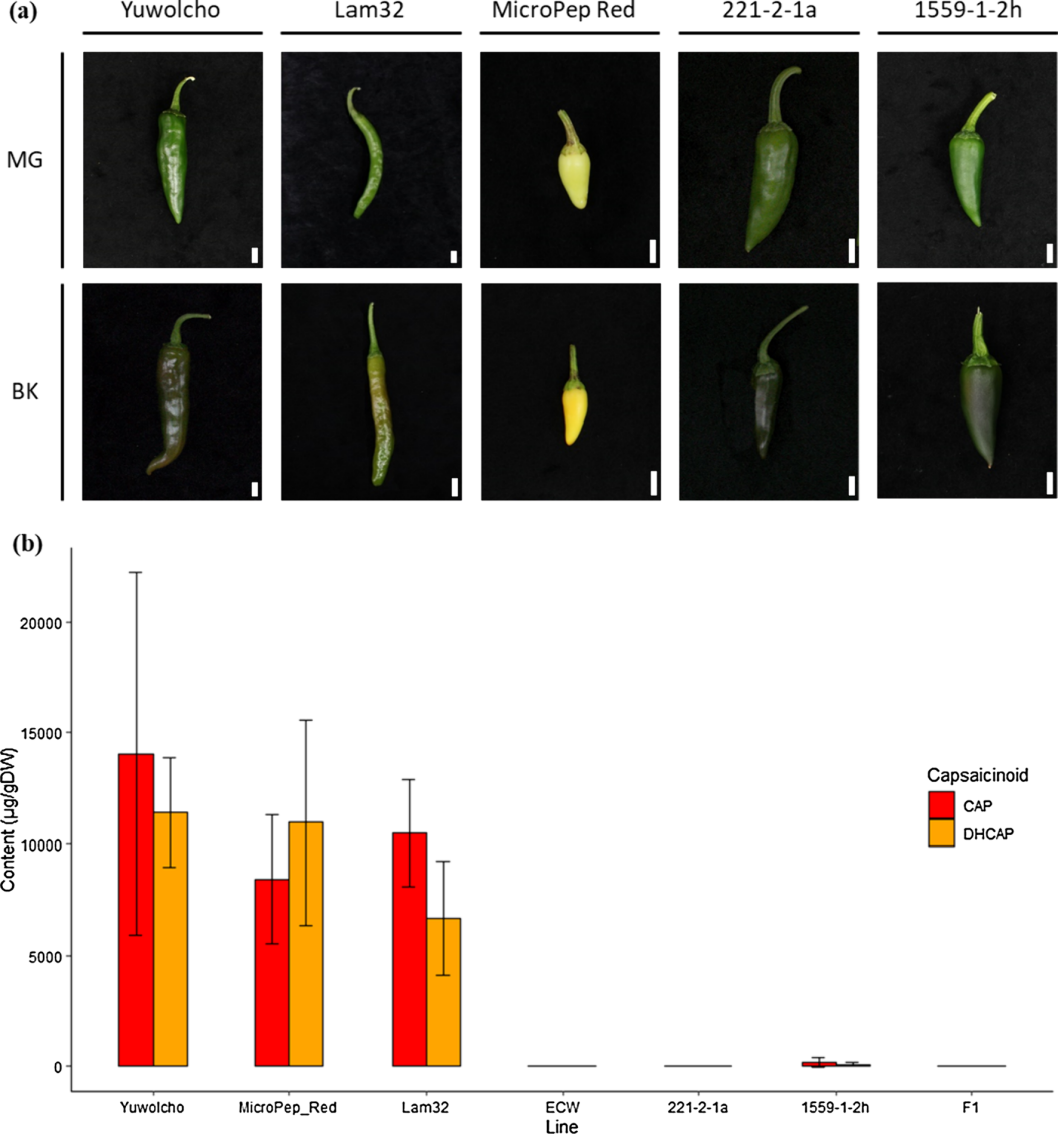
Extent of phenotypic variation among pepper fruits used in this study. **a** Fruit morphologies of parental and control lines. MG, mature green stage; BK, breaker stage. Scale bars, 1 cm. **b** Capsaicinoid content of parental and control fruits. Yuwolcho, MicroPep_Red, and Lam32 are pungent cultivars; ECW is a non-pungent cultivar. F1, hybrid of 221-2-1a and 1559-1-2h. Capsaicinoid content is shown as capsaicin (CAP) and dihydrocapsaicin (DHCAP) concentrations in fruit placental tissues. At least three pepper fruits were used for replicated samplings. Values are means ± standard deviation (SD)

The *Capsicum annuum* accessions ‘Yuwolcho’ (wild type: WT), ‘Lam32,’ and ‘Micro-Pep Red’ (MR) were selected from the germplasm of the Horticultural Crops Breeding and Genetics Laboratory (Seoul National University, Seoul, Republic of Korea) (Fig. 1a). The *C. annuum* mutant lines ‘221-2-1a’ and ‘1559-1-2h’ showing extremely low pungency derived from an ethyl methanesulfonate (EMS)-induced Yuwolcho mutant population, were selected to construct three populations in this study (Hwang et al. 2014; Jeong et al. 2012a). The first population, ‘221Y,’ consisting of 133 F_2_ and 80 F_3_ plants, was derived from a cross between 221 and 2-1a and WT. The bulked samples of 18 low-pungent F_3_ lines selected from the 221Y population and 18 WT plants were used for bulked-segregant analysis by sequencing (BSA-seq). The second population, ‘221L,’ was derived from a cross between 221 and 2-1a and Lam32. A total of 283 F_2_ plants were obtained to delimit the candidate gene interval. The third population, ‘1559M’, was derived from a cross between 1559 and 1-2h and MR. Among the 136 F_2_ plants, 36 plants (18 pungent and 18 with low pungency) were selected for bulked-segregant RNA sequencing (BSR-seq).

Allelism tests were conducted by crossing 1559-1-2h to *C. annuum* ‘ECW’, *C. annuum* ‘YCM334’, and *C. chinense* ‘No. 3341.’ Another test was conducted by crossing 221-2-1a to 1559-1-2h. The F_1_ plants from each cross were grown to evaluate the capsaicinoid content of their placental tissues.

### Capsaicinoid content evaluation

Capsaicinoid content was evaluated using the Gibb’s reagent test and high-performance liquid chromatography (HPLC). Capsaicinoid extracts were prepared from at least three fruits at the mature green (MG) and breaker (BK) stages for each plant following the method of Han et al. (2013). For the capsaicinoid content of the 221Y F_2_, F_3_, and 221L F_2_ populations, Gibb’s reagent method was used to determine the presence of capsaicinoids in plants (Jeong et al. 2012b). Those fruits that contained no capsaicinoids based on the Gibb’s reagent test were re-analyzed by HPLC. In the case of the 1559M F_2_ population, all 136 F_2_ plants were analyzed by HPLC. The HPLC analysis was performed at the National Instrumentation Center for Environmental Management (NICEM; Seoul, Republic of Korea). Total capsaicinoid content was calculated as the sum of capsaicin and dihydrocapsaicin contents, and the phenotype (pungent or non-pungent) was determined from comparison to total capsaicinoid content in the control and parental lines each year.

### Capsaicinoid biosynthetic gene (CBG) expression analysis

The expression of CBGs was analyzed by calculating the Log_2_ fold-change between the expression estimates for WT and 221-2-1a, using fragments per kilobase of transcripts per million mapped reads (FPKM) values derived from RNA sequencing (RNA-seq) analysis. Two separate RNA pools were obtained from at least three fruit placentas at 25 days post-anthesis (DPA) from seven plants. Total RNA was extracted from each plant using TaKaRa MiniBEST Plant RNA Extraction Kit (Takara Korea Biomedical, Seoul, Korea), and library construction and data analysis were followed the method of Byun et al. (2022) based on a NovaSeq 6000 instrument (Illumina, San Diego, CA, US). A total of 73 loci corresponding to 48 CBGs were selected from the *C. annuum* ‘CM334’ v1.6 genome based on their sequence similarity with genes annotated as capsaicin biosynthesis-related genes in previous studies (Kim et al. 2017; Mazourek et al. 2009). Among the CBGs, eight genes, *Pun1* (*AT3*), *Pun3* (*CaMYB31*), *pAMT*, *BCAT* homolog, *BCKDH E1α* homolog, *KAS* homolog, *ACL* homolog, and *FatA* homolog, were analyzed by reverse-transcription quantitative PCR (RT-qPCR) at five stages of fruit development (14-, 21-, 28-, 35-, and 42-DPA) for the confirmation of RNA-seq data. The RT-qPCR was performed with the same condition as described by Byun et al. (2022) using Rotor-Gene 6000 real-time PCR instrument (Qiagen, Hilden, Germany) and *Actin* was used as a reference gene for RT-qPCR.

### MutMap analysis

MutMap analysis was carried out based on the whole-genome sequencing of a low-pungency bulk consisting of 18 F_3_ individuals derived from the 221Y population and a WT bulk from 18 WT plants. Two separate paired-end DNA libraries were constructed for WT and 18 F_3_ samples using a TruSeq DNA PCR-Free kit (Illumina, San Diego, CA, US) as per Illumina guidelines. The libraries were sequenced on an Illumina HiSeq 4000 instrument, producing paired-end reads of 100 bp. Based on the whole-genome sequencing data of WT, a Yuwolcho reference genome was constructed by replacing the polymorphic positions in the *C. annuum* Dempsey genome (Lee et al. 2022) with their corresponding nucleotides obtained from Illumina short reads of Yuwolcho. MutMap analysis was conducted using the MutMap pipeline version 1.4.4 from Iwate Biotechnology Research Center (IBRC; http://genome-e.ibrc.or.jp) (Abe et al. 2012). Coval software was used to filter low-quality reads based on the number of mismatches, and SNP indices were calculated at all SNP positions (Kosugi et al. 2013). The SNP-index plots were plotted in R. The effects of SNPs were annotated with SnpEff (Cingolani et al. 2012).

### Genetic mapping of *Pun4*

A total of 153 genome-wide SNP markers distinguishing between WT and Lam32 were selected from 412 SNP marker sets, which have been designed for marker-assisted backcrossing (MABC) (Kang et al. 2014), to construct a linkage map of the 221L F_2_ population, consisting of 283 plants. The genetic map was constructed using a minimum logarithm of the odds (LOD) score of 3 and a maximum genetic distance of 30 centimorgan (cM) between consecutive markers using CARTHA GENE software (de Givry et al. 2005). For fine-mapping of *Pun4* on chromosome 6, a systematic approach was followed, leading to the development of 11 high-resolution melting (HRM) markers (Table S1). For HRM, a Roter-Gene 6000 real-time PCR instrument (Qiagen, Hilden, Germany) and Rotor-Gene Q series software version 2.1.0 were used. To determine the physical locations of the markers, marker sequences were compared to the Dempsey, CM334 v1.6, and ‘Zunla-1’ v2.0 genomes by BLASTn (Kim et al. 2017; Lee et al. 2022; Qin et al. 2014).

### De novo contig level assembly of Yuwolcho genome

High-quality genomic DNAs obtained from Yuwolcho leaf tissues were subjected to sequencing on the Pacific Biosciences (PacBio, CA, USA). Single-molecule real-time (SMRT) sequencing platform. The resulting high-fidelity (HiFi) reads were then de novo assembled into contigs using hifiasm (Cheng et al. 2021), and the quality of the assembly was assessed through benchmarking universal single-copy orthologs (BUSCO) analysis (Solanales odb10) at the National Instrumentation Center for Environmental Management (NICEM; Seoul, Republic of Korea). Following the identification of SNP marker positions used in genetic mapping on the assembled contigs, local gene prediction was carried out for a 6-Mb region within a contig near the *Pun4* locus using MAKER (Cantarel et al. 2008). The predicted genes were subsequently functionally annotated through a BLASTp search against the Swissprot plant protein database.

### Detection of differentially expressed genes (DEGs) and gene ontology (GO) enrichment analysis

DEGs were identified from the RNA-seq reads of WT and 221–2-1a and two separate RNA pools from the 1559M F_2_ population. In the case of WT and 221–2-1a, the same samples used for the CBG expression analysis above were analyzed, and the RNA pools of the 1559M F_2_ population were obtained from the placental tissues of at least three fruits at 25-DPA from 18 with the highest capsaicinoid content and 18 plants with the lowest capsaicinoid content, respectively. These two groups were then divided into three pools each, and RNA extraction, library construction, and sequencing of two separate RNA pools from the 1559M F_2_ population were conducted in the same manner as described earlier. DEGs between WT and 221–2-1a or the 1559M F_2_ pools were identified with the DESeq2 R package using RNA-seq reads aligned to the ‘Dempsey’ reference genome (Lee et al. 2022; Love et al. 2014). Among the DEGs, CBG homologs were annotated and their expression levels visualized using the EnhancedVolcano R package (Blighe et al. 2019). GO term enrichment analysis of DEGs was performed using agriGO v2.0 and the GO terms were grouped into higher groups using REVIGO (Supek et al. 2011; Tian et al. 2017). For agriGO v2.0, annotated GO terms were analyzed with single enrichment analysis (SEA); Fisher method was used as statistical test.

### Weighted gene co-expression network analysis (WGCNA)

A gene co-expression network analysis was conducted using the R package WGCNA to identify genes whose expression pattern correlated with that of differentially expressed CBGs between WT and 221-2-1a (Langfelder and Horvath 2008). FPKM values for five different stages of WT placenta development (14-, 21-, 28-, 35-, and 42-DPA) were used for this analysis. The FPKM values for seven CBGs (*BCAT*, *BCKDH E1α*, *ACL*, *KasI*, *KasIII*, *FatA*, and *AT3*) that showed differential expression between WT and 221-2-1a were used as trait data input. Adjacency (network connectivity) values between each gene expression pattern were calculated using a ‘signed’ network type and a soft threshold power value of 65. Adjacency values were then translated into a topological overlap matrix (TOM) to calculate the corresponding dissimilarity (dissTOM = 1 − TOM). Gene clustering was performed based on dissTOM, and a tree plot was drawn using the cutreeDynamic function, with each branch displaying modules of clustered eigengenes (MEs). The correlation between MEs and trait data was calculated, and a correlation heatmap was drawn according to this calculation.

### Quantitative trait locus (QTL)-seq analysis

QTL-seq analysis of the 1559M F_2_ population was conducted using the QTLseqr package (Mansfeld and Grumet 2018). The same data of two RNA pools from 1559M F_2_ population that used in DEG analysis were used. The RNA reads were aligned to the *C. annuum* Dempsey genome, and the significant single-nucleotide polymorphisms (SNPs) between the two groups were selected and filtered through the QTLseqr package.

## Results

### Characterization of two EMS mutants with extremely low pungency

We characterized the two extremely low-pungent mutant lines 221-2-1a and 1559-1-2h, derived from an EMS-mutagenized population in the Yuwolcho cultivar, to identify the genetic factor (s) that regulate fruit pungency. We performed HPLC analysis of placental tissues, which revealed that the 221-2-1a and 1559-1-2h mutants accumulated 17.7 ± 24.8 μg/g dry weight (DW) and 258.8 ± 285.7 μg/g DW of total capsaicinoids, respectively, much less than did the parental and control lines (20,979.2 ± 8195.5 μg/g DW) (Fig. 1b). To determine whether 221-2-1a and 1559-1-2h harbor a mutation in the same gene, we crossed the two mutants and measured the capsaicinoid content of their F_1_ progeny, using the placenta (Table 1). All these F_1_ plants had extremely low pungency, as evidenced by an average capsaicinoid content of 34 μg/g DW. We thus conclude that the mutants 221-2-1a and 1559-1-2 h carry a mutation in the same gene.

**Table 1:** Summary of allelism tests between mutants and non-pungent accessions. All values are for the F_1_ progeny of the indicated parental lines

	*C. annuum* ECW (*pun1/pun1*)	*C. annuum* YCM334 (*pun3/pun3*)	*C. chinense* No. 3341 (*cakr1/cakr1*)	*C. annuum* Micro-Pep Red (wild type)	*C. annuum* 221–2-1a
*C. annuum* 1559–1-2 h	3,383^a^ ± 1,420^b^ (*n* = 3) (*p* = 0.028)^c^	4,687 ± 996 (*n* = 3) (*p* = 0.007)	36,996 ± 3,469 (*n* = 3) (*p* = 0.001)	13,125 ± 1297 (*n* = 3) (*p* = 0.022)	33 ± 17.0 (*n* = 12)

^a^Average of total capsaicinoid concentrations of the mature green stage placenta in F_1_ plants (μg/g dry weight)

^b^Standard deviation for each group

^c^*p* value for t-test with F_1_ between the mutants

### Inheritance of *Pun4*, a novel locus controlling pungency in Capsicum

We generated three segregating populations to identify the causal mutation behind the low-pungency phenotype of the mutants 221-2-1a and 1559-1-2 h. More precisely, we crossed 221-2-1a to the cultivar Yuwolcho (221Y population) or to the cultivar Lam32 (221L population); we also crossed 1559-1-2 h to the cultivar Micro-Pep Red (1559 M population). We evaluated the pungency of each plant based on Gibb’s reagent and HPLC analysis. Gibb’s reagent produced a blue color, which we compared to serially diluted standards to estimate capsaicinoid content. We considered a capsaicinoid content below 3,000 μg/g DW through HPLC analysis as low pungency, based on the distribution of capsaicinoid content within the 1559 M F_2_ population (Fig. S1). All F_1_ plants from all three crosses had pungent fruit, indicating that the two mutations are recessive. Pungency exhibited distinct segregation ratios in various F_2_ populations (Table 2). The segregation ratio for the 221L F_2_ and the 1559M F_2_ populations was 4:1 and 2:1, respectively, suggesting that the low-pungency phenotype of the mutants is caused by the mutation of one major gene with additional influence from minor factors.

**Table 2:** Summary of plant materials used in this study

Population	Number of plants			Ratio (Pungent:Low-pungency)	Phenotyping method
	Pungent	Low-pungency	Total		
221Y population					
221–2-1a × Yuwolcho F_1_	5	0	5	1:0	Gibb's reagent analysis (MG stage placenta)
221L population					
221–2-1a × Lam32 F_1_	9	0	9	1:0	Gibb's reagent analysis & HPLC (MG and BK stage placenta)
221–2-1a × Lam32 F_2_	230	53	283	4:1 (*p* = 0.5926)	
1559 M population					
1559–1-2 h × Micro-Pep Red F_1_	5	0	5	1:0	HPLC (MG stage placenta)
1559–1-2 h × Micro-Pep Red F_2_	90	46	136	2:1 (*p* = 0.9034)	

*MG* mature green stage *BK* breaker stage *HPLC* high-performance liquid chromatography

### Allelism test with *pun1*, *pun3*, and *cakr1* mutants

The genes *Pun1, Pun3, pAMT,* and *CaKR1* contribute to the control of capsaicinoid biosynthesis in *Capsicum* (Han et al. 2019; Koeda et al. 2019; Lang et al. 2009; Stewart Jr et al. 2005). We sequenced these genes in the mutants 221-2-1a and 1559-1-2 h but detected no mutations compared to their reference sequences. To validate these results, we conducted allelism tests by crossing the 1559-1-2 h mutant to *Capsicum* accessions harboring non-functional alleles of *Pun1*, *Pun3*, or *CaKR1*. All F_1_ hybrids from crosses between 1559 and 1-2h and ECW (*pun1/pun1*), 1559-1-2h and YCM334 (*pun3/pun3*), and 1559-1-2h and No. 3341 (*cakr1/cakr1*) had significantly pungent fruit, with an average capsaicinoid content over 3,000 μg/g DW (Table 1). These results suggest that the low-pungent mutants 221-2-1a and 1559-1-2 h harbor functional alleles of *Pun1*, *Pun3*, and *CaKR1*. Therefore, we named the locus defined by these two mutants as *Pun4*.

### Expression of CBGs in WT and 221–2-1a

**Fig. 2:**
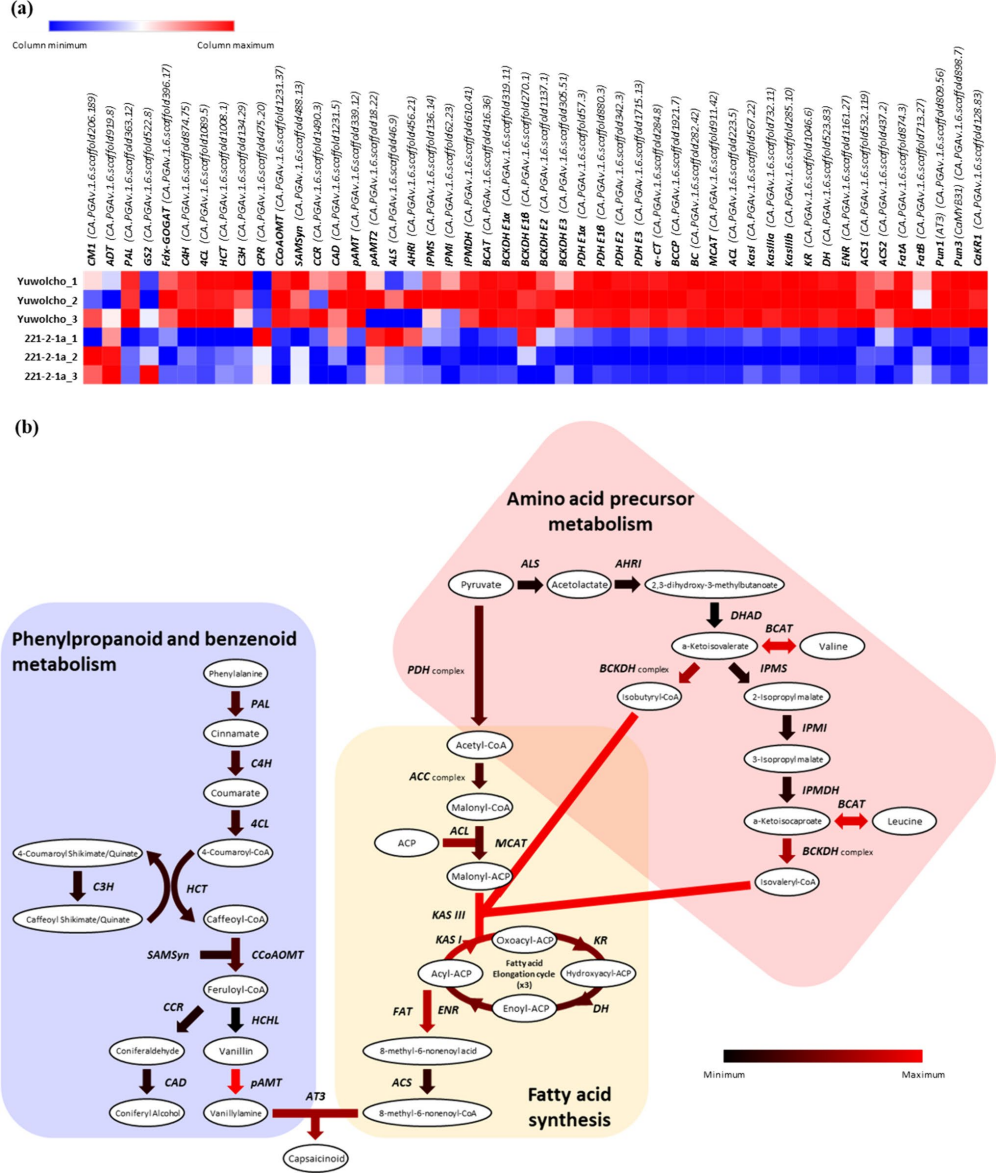
Expression of CBGs in WT and the mutant 221-2-1a. **a** Heatmap representation of the expression levels of CBGs in WT and 221-2-1a. Values are shown as Log_2_(FPKM + 1). **b** Overview of the capsaicin biosynthesis pathway. Biosynthetic intermediates are shown in white ellipses, with the names of each structural gene encoding the enzyme responsible for the step shown by the arrows. The expression values are shown as Log_2_([FPKM of WT / FPKM of 221-2-1a] + 1), with black arrows indicating no difference between the two genotypes and red indicating higher expression in WT. Most of the genes showing decreased expression in the mutant, such as *BCAT*, *BCKDH*, *ACL*, *MCAT*, *KasI*, *KasIII*, and *FAT*, belong to the latter part of valine/leucine metabolism and fatty acid biosynthesis pathways. Thee pathways were modified from Mazourek et al. (2009)

We identified 73 CBG-like genes based on their sequence similarity to genes annotated as capsaicin biosynthesis-related genes in previous studies (Kim et al. 2017; Mazourek et al. 2009). Of these 73 genes present in the CM334 v1.6 reference genome, we narrowed down the list to 48 CBGs that are expressed in the placentas of 25-DPA fruit and determined their expression levels in WT and 221-2-1a by RNA-seq (Table S2, Fig. 2a). Among the 48 CBGs, 42 gene were expressed to lower levels in the 221-2-1a mutant compared to WT. The expression of functionally identified genes, such as *Pun1* (*AT3*), *Pun3* (*CaMYB31*), *pAMT*, and *CaKR1*, was much lower in 221-2-1a relative to WT, as was the expression of genes associated with the branched-chain fatty acid biosynthesis pathway, including *BCAT*, *BCKDH*, *ACL*, *MCAT*, *KasI*, *KasIII*, and *FAT*. By contrast, we observed minor differences in the expression levels of genes involved in the phenylpropanoid pathway (Fig. 2b). We validated the RNA-seq analysis by conducting RT-qPCR on eight genes using RNA samples extracted from five different stages of fruit development (Fig. S2). All genes showed a significant decrease in expression in the mutant compared to the WT throughout the various stages.

### Genetic mapping of *Pun4* with genome-wide SNP markers

To determine the chromosomal location of the *Pun4* locus, we constructed a linkage map based on 89 F_2_ plants from the 221L F_2_ population and 153 genome-wide SNP markers that are polymorphic between WT and Lam32. We obtained 12 linkage groups, using a maximum genetic distance between consecutive markers of 30 cM, resulting in a map with an average genetic distance of 6.8 cM between consecutive markers (Fig. S3a). We then used this genetic map to determine the location of the *Pun4* locus, using the pungency phenotype of the 221L F_2_ population. We mapped the *Pun4* locus to the middle of the linkage group LG6, between the markers CAPS_CONTIG.192 and KS20039B04, defining a genetic interval of 12.3 cM and a physical interval ranging from 209.05 Mb to 210.96 Mb on the chromosome 6 (Fig. S3b).

### MutMap analysis for the detection of EMS-induced causal SNPs

We independently performed a MutMap analysis to identify sequence differences between WT and the mutant 221-2-1a that would be consistent with an EMS-induced mutation. We performed BSA-seq of two pools, one containing genomic DNA of WT fruits and one from F3 individuals of the 221Y population with low pungency. Of the clean reads produced from sequencing of these two pools, 91.7% and 68.5% of reads aligned to the Dempsey reference genome, respectively (Table S3). We then generated a pseudogenome for Yuwolcho by introducing the Yuwolcho-type SNPs into the Dempsey reference genome, followed by MutMap analysis with a 4-Mb sliding window size and 10-kb increment. Of the 12 chromosomes, we observed consecutive peaks with ΔSNP-indices above the 99% confidence interval of the sliding window average only for chromosome 6 (Fig. S4). We detected four peaks spanning 193.11–215.89 Mb on chromosome 6 with ΔSNP-indices of 1. Within these peak regions, we identified 2,560 SNPs, of which 216 potential sequence differences were consistent with an EMS mutation (G-to-A and C-to-T conversions) with a mutant allele frequency (AF) exceeding 0.6. Of these 216 sequence differences, we retained 41 based on their location within the 208–212-Mb region encompassing the mapping interval defined above (Fig. S5).

### Fine-mapping of the *Pun4* locus

**Fig. 3:**
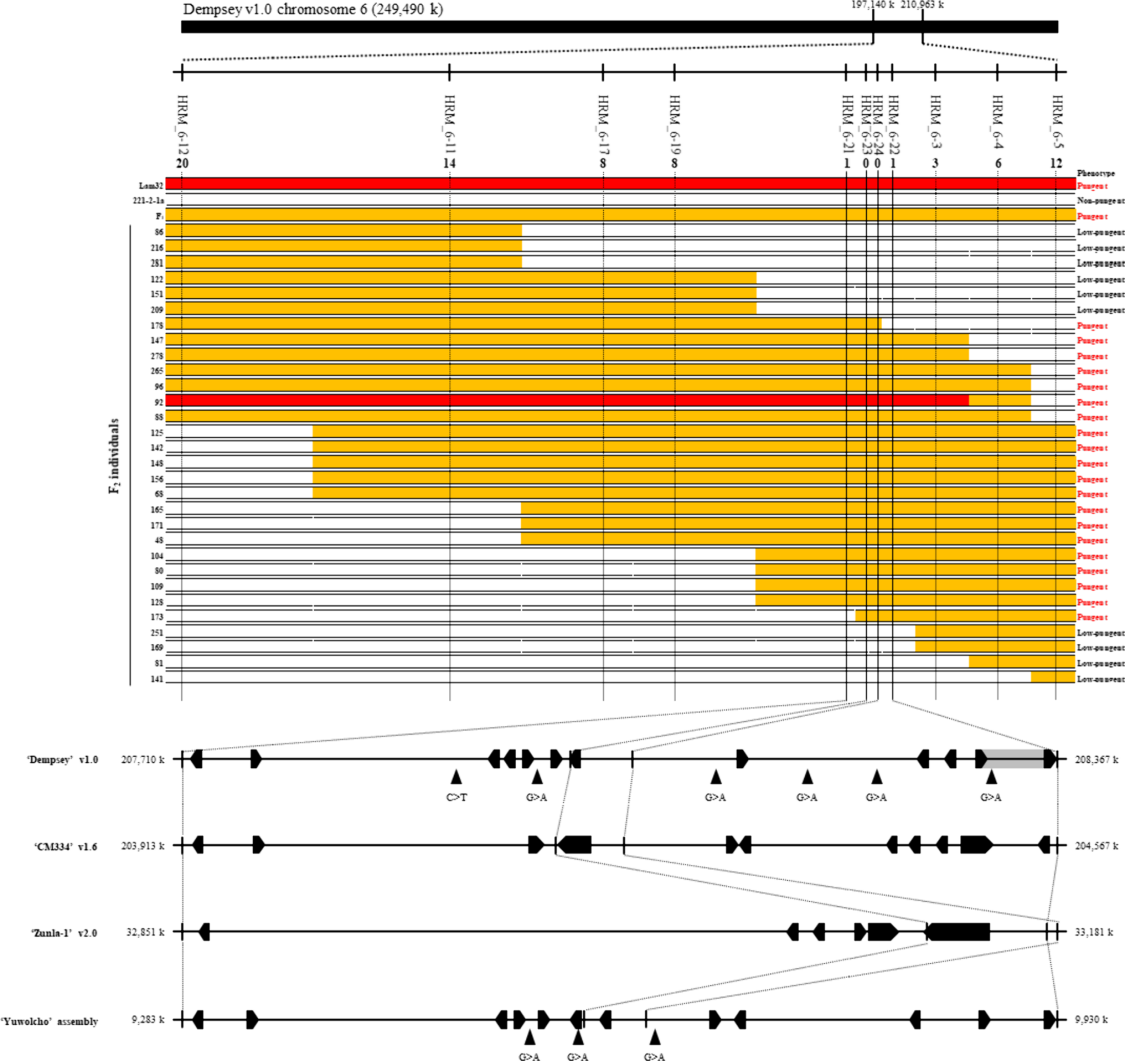
Fine-mapping of candidate locus on chromosome 6. Using 11 HRM marker sets in the mapping interval, the genotypes of 30 recombinants with different pungency phenotypes were used for genotyping. Red, pungent (Lam32 genotype); white, low-pungency phenotype (221-2-1a genotype); orange, pungent phenotype (heterozygous). The number of recombinants for each marker is given below the marker name. The black arrowheads denote EMS-induced SNPs in each genome sequences over this interval. Annotated genes are shown as black arrows in each reference genomes. The gray box shows a misannotated region

To further delimit the mapping interval of *Pun4*, we designed 11 HRM markers to genotype 283 F_2_ plants from the 221L population. Of these 283 plants, we selected 30 recombinants, each exhibiting various recombination patterns within the mapping interval of 197.14–210.96 Mb on chromosome 6 (Fig. 3). We narrowed down the target region to a 66-kb interval through fine-mapping, from 207.71 to 208.37 Mb. In the Dempsey reference genome, this interval contains 11 genes and six sequence differences, based on the MutMap analysis. Only one of these differences was within a large intron within DEMF06G16330, based on the current genome annotation (Fig. 3) (Table S4). However, it is likely an intergenic region due to the misannotation caused by the high similarity between two genes (Fig. S6). We mapped all six EMS-type sequence differences onto the CM334 v1.6 and Zunla-1 v2.0 genomes, but no consensus was found regarding their location within the genic region or the 3-kb promoter region.

### De novo contig level assembly of Yuwolcho genome

The genome of Yuwolcho was sequenced and assembled at the contig level using long reads from PacBio SMRT sequencing. A total of 5,141,472 HiFi reads were generated, resulting in approximately 70.8 Gb (about 23 × coverage) of read bases. The contig assembly produced 726 contigs, which were subsequently filtered to 244 contigs originating from organelles. The assembly process notably increased the N50 value (the size of the shortest contig fragment representing 50% of the total genome) from 14.06 kb to 95.29 Mb (Table S5), and BUSCO analysis indicated a high completeness of 98.2% for the assembly (Table S6). The contig labeled 'ptg000019l' contained HRM markers tightly linked with the *Pun4* locus in genetic mapping (Table S1). Therefore, a 6-Mb region spanning approximately 205–211 Mb of the Dempsey reference genome was chosen for functional annotation. The functional annotation predicted a total of 173 proteins in this sequence, with 151 proteins annotated through BLASTp against the Swissprot plant protein database using a criterion of e-value < 1.

### Identification of DEGs and GO term enrichment analysis

**Fig. 4:**
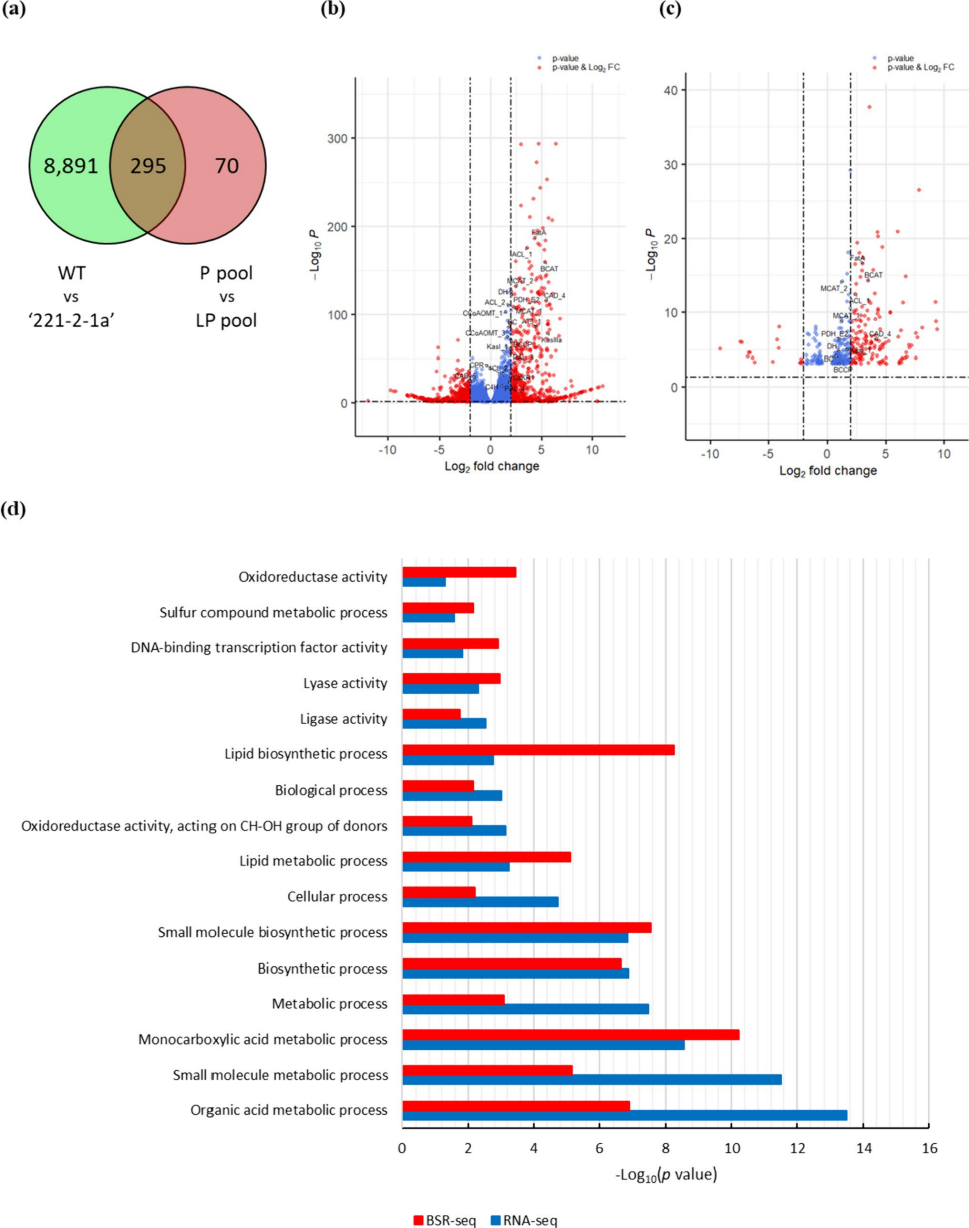
DEGs related to pungency. **a** Venn diagram showing the overlap between the number of detected DEGs between WT and the mutant 221-2-1a and between the pungent pool and the low-pungency pool derived from the 1559M F_2_ population using fruit placenta. **b, c** Volcano plot of DEGs between WT and 221-2-1a (**b**) and between the two 1559M F_2_ pools (**c**). Blue circles represent DEGs with an absolute Log_2_(fold-change) < 2; red circles represent DEGs with an absolute Log_2_(fold-change) > 2 and *p* value < 0.05. Putative CBGs were annotated using black arrows in the plot. **d** Enriched GO terms among DEGs common to the WT vs 221-2-1a comparison and to the two extreme 1559M F_2_ pools. Each value indicates the −Log_10_(*p* value) (color figure online)

To identify DEGs associated with the *Pun4* gene, we analyzed two datasets: specifically, we compared expression levels between WT and 221–2-1a, as well as between pools of pungent and low-pungency fruits from 1559M F_2_ individuals. We defined DEGs based on the criteria of *p* value < 0.05 and absolute Log_2_(fold-change) > 2. We identified 9186 DEGs between WT and 221-2-1a, of which 4448 were upregulated in the mutant and 4738 were downregulated in the mutant. Similarly, we identified 365 DEGs between the two 1559M pools, with 70 upregulated DEGs and 295 downregulated DEGs in the low-pungency pool. We discovered that nearly all CBGs were downregulated in the mutant and in the low-pungency pool, as illustrated by volcano plots (Fig. 4a–c). Comparison of the two datasets revealed 295 overlapping DEGs. For each set, we performed a GO term enrichment analysis using the SEA of AgriGO v2.0 and summarized the obtained GO terms by REVIGO, using a *p* value threshold of < 0.05. The two sets had 16 common GO terms (Fig. 4d), including ‘lipid biosynthetic process,’ ‘lipid metabolic process,’ ‘monocarboxylic acid metabolic process,’ and ‘organic acid metabolic process.’ These terms are related to lipid metabolism and may identify genes that serve in the steps of capsaicinoid biosynthesis.

### WGCNA using the expression of CBGs across fruit development

**Fig. 5:**
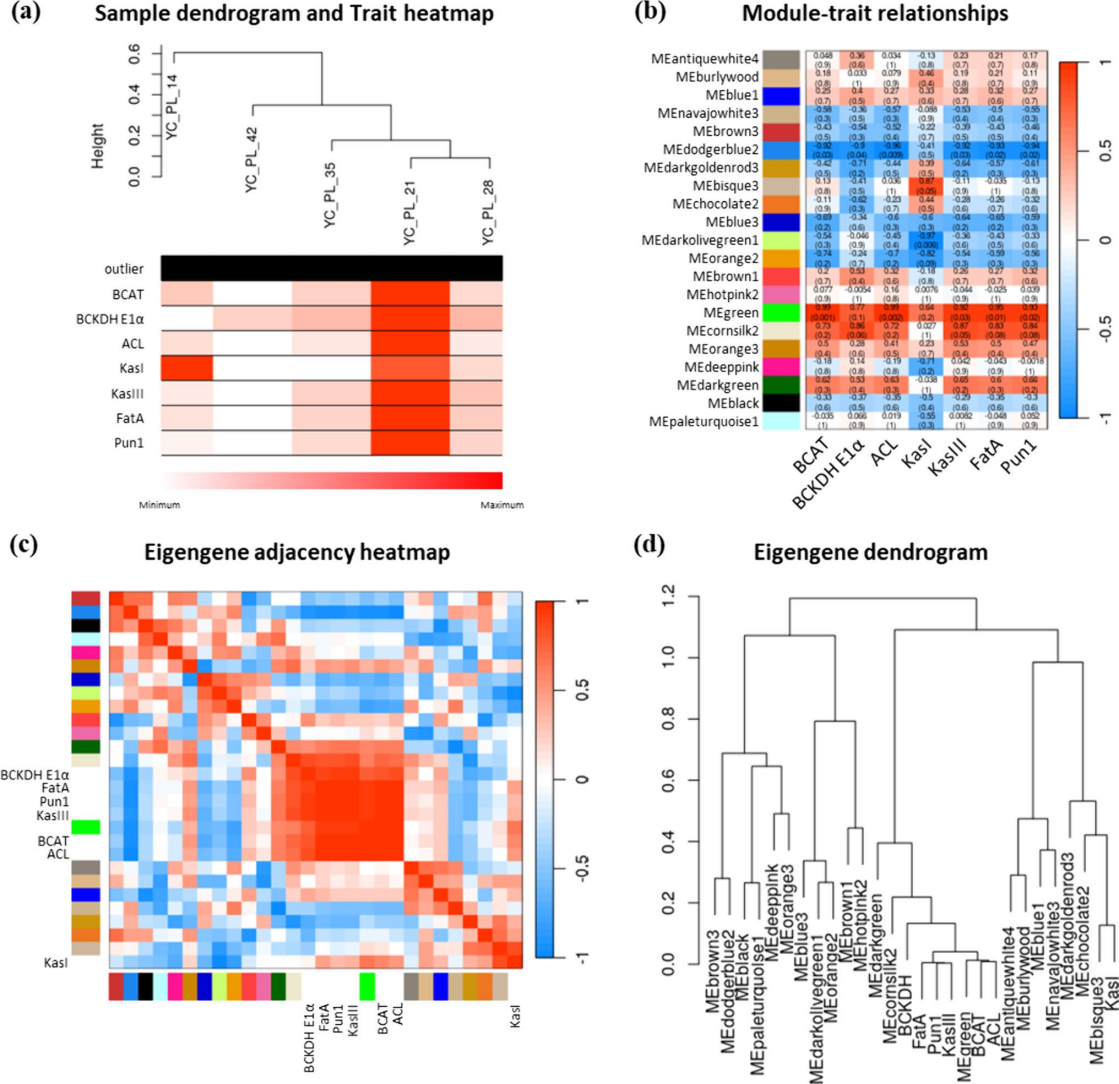
Summary of WGCNA. Twenty-one MEs were identified, each represented by a color name. The expression levels of *BCAT*, *BCKDH E1α*, *ACL*, *KasI*, *KasIII*, *FatA*, and *AT3* (*Pun1*) were used as trait input. **a** Dendrogram of samples based on the similarity in expression pattern of the seven CBGs. In the heatmap, intensity of red indicates higher expression. **b** Module–trait relationships, as indicated by Pearson’s correlation coefficients. **c, d** Correlation between MEs and traits, represented as heatmap (**c**) or dendrogram (**d**)

We performed WGCNA to refine the list of candidate genes showing similar expression patterns as seven CBGs that displayed significant differential expression between WT and the mutant among the identified DEGs. We determined gene expression levels in five pools of WT fruit tissues at different developmental stages and used these expression values to construct a co-expression network, resulting in 21 MEs. Among these, MEgreen showed the strongest correlation with the expression levels of the seven CBGs mentioned above between WT and the mutant (Fig. 5b). However, *KasI*, the only gene showing high expression in 14-DPA fruits (Fig. 5a), was highly correlated with MEbisque3. Although MEgreen was the most highly positively correlated ME with all seven CBGs (Fig. 5c), each of these CBGs exhibited significant correlations with different MEs, such as MEbisque3 (*ACL*, *KasIII*), MEcornsilk2 (*BCAT*), MEblack (*BCKDH E1α*), MEblue3 (*KasI*), and MEbrown1 (*Pun1*). From the co-expression network, we extracted 512 genes located on chromosome 6 that belong to these MEs for characterization.

### Integration of fine-mapping, BSA-seq, and RNA-seq to identify candidates

RNA-seq analysis and WGCNA helped us define 512 genes on chromosome 6 that are differentially expressed in the 221-2-1a mutant and whose expression is highly correlated with that of CBGs. Among these genes, 12 were located close to the final mapping interval (207.71–208.37 Mb). Of these 12 genes, we retained five as high-confidence candidate genes based their Log_2_(fold-change) values between WT and 221-2-1a (Table 3). We functionally annotated these five genes through BLASTp searches using the non-redundant (nr) database from the National Center for Biotechnology Information (NCBI) to predict gene function. Of these five genes, we speculate that DEMF06G16460 is a strong candidate for the *Pun4* locus, as its encoded protein shows similarity to 3-ketoacyl-CoA synthases, which may function as a long fatty acid synthase downstream of the fatty acid elongation cycle that could start the middle of capsaicinoid biosynthesis to bypass. It is also possible that one or more of the final six sequence differences we detected from MutMap analysis is the causal mutation and may interact with the candidate gene.

**Table 3:** List of final candidate genes from the combination of DEG analysis between WT and the mutant 221–2-1a, WGCNA, and genetic mapping

Module color	Gene	Position (bp)		Log_2_(fold-change)	Expression in WT placenta (FPKM)					Description
		Start	End		14 DPA	21 DPA	28 DPA	35 DPA	42 DPA	
MEbisque3	DEMF06G16090	207,373,472	207,375,274	− 1.53	6.9	7.3	6.8	8.95	19.0	Peptide-N4-(N-acetyl-beta-glucosaminyl)asparagine amidase A
MEblue3	DEMF06G16300	208,126,731	208,129,730	− 1.33	14.5	2.0	2.9	0.9	0.4	Serine/threonine protein phosphatase 2A 57-kDa regulatory subunit B' kappa isoform
MEbisque3	DEMF06G16380	208,646,659	208,646,931	3.97	0.5	5.3	1.7	2.45	1.05	RNase H type-1 domain-containing protein
MEblue3	DEMF06G16400	208,788,942	208,790,227	− 3.14	0.4	0.1	0.2	0.2	0.45	Putative LRR receptor-like serine/threonine-protein kinase-like
MEblue3	DEMF06G16460	209,044,421	209,045,959	− 1.44	167.3	223.0	213.3	236.5	164.1	3-ketoacyl-CoA synthase 11

*DPA* days post anthesis *FPKM* fragments per kilobase of transcript per million mapped reads

## Discussion

In this study, we characterized the two extremely low-pungency mutants 221-2-1a and 1559-1-2h from an EMS-mutagenized population and investigated the genetic characteristics of the causal *Pun4* locus across three segregating populations. The expression of CBGs in the 221-2-1a mutant suggests a potential association between *Pun4* and branched-chain fatty acid biosynthesis. Furthermore, we propose a strong candidate gene from genetic mapping and DEG analysis. Overall, we speculate that the newly identified *Pun4* locus is directly or indirectly involved in the biosynthesis of branched-chain fatty acids during capsaicinoid biosynthesis.

### Characterization of mutants in a new gene controlling capsaicin biosynthesis

The two mutants 221-2-1a and 1559-1-2h accumulate only 0.07% and 1.02%, respectively, of the capsaicinoid content in the WT. The mutant 221-2-1a almost completely lacks capsaicinoids, similar to ECW cultivar that harboring *Pun1* loss-of-function allele (Fig. 1b). An allelism test confirmed that the two mutants harbor a mutation in the same causal locus, *Pun4*; the reason for their phenotypic differences is unknown. However, a similar phenomenon was observed in a previous study of *pAMT* alleles, with weaker alleles being caused by the insertion of transposable elements at various positions within the same intron of the *pAMT* gene (Tanaka et al. 2019).

All F_1_ plants derived from crosses between the mutant 1559-1-2h and non-pungent varieties (*pun1*, *pun3*, and *cakr1*) accumulated significantly more capsaicinoids than did the mutants (Fig. 1b) (Table 1), indicating that the *Pun4* locus is unlikely to be allelic to these known genes. The much higher capsaicinoid content of F_1_ plants derived from the cross 1559-1-2h × No. 3341 (*cakr1/cakr1*) compared to the wild-type *C. annuum* accessions used in this study may reflect the high pungency of *C. chinense*, which was used as the paternal line. All F_1_ plants derived from crosses between each mutant (221-2-1a and 1559-1-2h) and pungent cultivars were also pungent (Table 2), suggesting that the *Pun4* locus is dominant, similar to other functionally identified CBGs such as *Pun1*, *Pun3*, *pAMT*, and *CaKR1* (Han et al. 2019; Koeda et al. 2019; Lang et al. 2009; Stewart Jr et al. 2005).

A combined analysis of linkage mapping and BSA-seq anchored the *Pun4* locus to chromosome 6 around 208-Mb in the Dempsey reference genome, indicating one major gene (Fig. 3) (Fig. S5). However, the segregation ratios of two different F_2_ populations were inconsistent (Table 2), suggesting the segregation of one major dominant gene (*Pun4*) together with minor contributing factor(s). While pungency was treated as a qualitative trait, the strength of pungency is a quantitative trait that is affected by various factors, including environmental factors such as light, temperature, water availability, or nutrition (Naves et al. 2019). Therefore, further research is required to identify the specific factors contributing to the variation in pungency in the segregating populations.

### Selection of candidate genes for CBGs based on transcriptome analysis

We conducted RNA-seq analysis of two datasets (between WT and 221-2-1a; between individuals from the 1559M F_2_ population with high pungency or low pungency). Independently, we measured gene expression in WT fruits across five developmental stages; we used these results as input for WGCNA and looked specifically for genes that are co-expressed with known or suspected CBGs. In the DEG analysis, we identified 9186 DEGs and 365 DEGs, respectively (Fig. 4a), with most CBGs being downregulated in the mutant (221-2-1a) and the low-pungency pool (Fig. 4b, c). Among the downregulated DEGs in 221-2-1a relative to its WT, genes showing at least a four-fold difference in expression included genes similar to *BCAT*, *BCKDH*, *PYRUVATE DEHYDROGENASE* (*PDH*), *ACL*, *MCAT*, *KasIII*, *KasI*, *HYDROXYL-ACP DEHYDRATASE* (*DH*), and *FatA*, which are thought to be associated with fatty acid biosynthesis, with the exception of a *CINNAMYL ALCOHOL DEHYDROGENASE* (*CAD*)-like gene and the *AT3* gene (*Pun1*). Among the 11 genes, 5 (*BCAT*, *BCKDH*, *ACL*, *FatA*, and *CAD*) were common to both datasets, including DEMF06G16460, our candidate gene for *Pun4*. Based on these results, we hypothesize that *Pun4* is involved in fatty acid biosynthesis. The changes in fatty acid biosynthetic gene expression may be correlated with the downregulation of the *CAD* homolog and the *AT3* gene, which are located at the end of the phenylpropanoid pathway. Therefore, all these CBG homologs should be functionally investigated: *BCAT* (DEMF04G22720), *BCKDH* (DEMF06G13250), *ACL* (DEMF01G33980), *FatA* (DEMF06G29890), and *CAD* (DEMF02G11700).

WGCNA produced 21 distinct MEs, of which MEgreen showed high correlation with the expression levels of seven CBGs that were DEGs in the above analysis (Fig. 5a–c). However, each CBG belonged to distinct MEs, prompting us to select all MEs that contained at least one CBG. We therefore selected 746 (MEbisque3), 241 (MEcornsilk2), 51 (MEblack), 615 (MEblue3), and 61 (MEbrown1) genes. Of these genes, 20 mapped onto chromosome 6 and were among the DEGs in dataset 1 and 2. After comparing their expression patterns to those of seven CBGs, we retained 15 genes as candidates (Table S7). We functionally annotated these genes, revealing CBG-like genes (DEMF06G11720, DEMF06G17360, DEMF06G29890, and DEMF06G30030) and a transcription factor gene (DEMF06G23780). These genes may also contribute to capsaicin biosynthesis, and their function should be examined in detail.

### QTL-seq analysis exhibits consistent results with other analyses

We conducted a BSR-seq analysis on two extreme pools derived from the 1559M F_2_ population to detect EMS-induced mutations within the mapping interval. While several chromosomes had strong discrete signals for Gprime analysis, only chromosome 6 exhibited consistently high Gprime values across the chromosome. Moreover, the defined interval overlapped with that obtained by genetic mapping (Fig. S7a). In the region of chromosome 6 after 100-Mb, we detected 1,245 EMS-induced mutations (comprising G-to-A and C-to-T conversions). We also detected several peaks exceeding the significance threshold on chromosomes 1, 2, 3, 4, 5, 7, 10, and 12, which likely reflect natural variation between WT and MR. Several of the Gprime peaks had a matching peak from QTL-seq analysis, although only two (on chromosomes 2 and 6) showed a ΔSNP index above the 99% confidence interval (CI) (Fig. S7b). We then filtered EMS-induced mutations located within the peak on chromosome 6, using 99% CI, leading to 338 sequence differences consistent with an EMS mutation. Of these, 12 were located in genic regions within the broad 208–212-Mb mapping interval. However, Sanger sequencing revealed that all of these differences could be attributed to polymorphisms between WT and MR rather than to EMS treatment. These findings indicate that the low-pungency phenotype of the mutant may not be attributed to sequence variation within genic regions.

### Lower expression of fatty acid pathway-related genes results in low pungency

The capsaicinoid biosynthetic pathway consists of two main branches (Fig. 2b), each sharing certain components with other pathways. Phenylpropanoid metabolism might be connected to various secondary metabolite pathways, including the shikimate, quinate, flavonoid, and lignin pathways (Fraser and Chapple 2011). MYB24, a negative co-regulator of capsaicin and lignin biosynthesis, might play a role behind this connection of pathways (Yu et al. 2023). Fatty acid biosynthesis is, however, thought to be more complex. It initiates with acetyl-CoA, which serves as the precursor for the citrate cycle, and involves several branching pathways for biosynthesis. These biochemical steps include elongation cycles that can generate a multitude of fatty acid analogs (Mazourek et al. 2009). To date, no research has explored the interaction or co-regulation between the capsaicinoid pathway and the fatty acid cycle. We determined that fatty acid biosynthetic genes are downregulated in fruits with low pungency, suggesting a connection between fatty acid biosynthesis and the capsaicinoid pathway (Figs. 2, 4b, c ). Among the genes identified as candidates for *Pun4*, the candidate gene DEMF06G16460 encodes a putative 3-ketoacyl-CoA synthase potentially associated with fatty acid elongation and wax biosynthesis (Table 3) (Todd et al. 1999). Hence, we propose that *Pun4* likely corresponds to this *KCS* homolog. KCS may play a role in a regulation of the production of a capsaicin pathway precursor and the regulation of long-chain fatty acids.

### Comparison to previously studied pungency-related loci on chromosome 6 of pepper

According to previous studies, the four capsaicinoid biosynthesis genes *Pun1*, *Pun3*, *pAMT*, and *CaKR1* are located on pepper chromosomes 2, 7, 3, and 10, respectively, based on the *C. annuum* genomes (Han et al. 2019; Koeda et al. 2019; Lang et al. 2009; Stewart Jr et al. 2005). In addition, various quantitative trait loci (QTLs) or genomic regions associated with capsaicinoid content in placenta or pericarp have been identified (Table 4) (Ben-Chaim et al. 2006; Blum et al. 2003; Cao et al. 2022; Han et al. 2018; Kondo et al. 2023; Lee et al. 2016; Yarnes et al. 2013). Among these genomic regions, two (*SJ-dhc6* and *punv*) are located near *Pun4*. *SJ-dhc6* was identified from a population derived from a cross between the non-pungent *C. chinense* cultivar ‘SNU11-001’ and the pungent *C. chinense* cultivar ‘Bhut Jolokia’ for capsaicinoid content in both placenta and pericarp. *punv* was identified by genome-wide association study (GWAS), with two polymorphisms in a gene (Capana06g001204) associated with pungency across a population of 347 cultivars from 12 *Capsicum* species (Cao et al. 2022; Park et al. 2019). We observed no sequence variation and expression difference that would alter the protein encoded by Capana06g001204 between WT and the *pun4* mutants. Therefore, Capana06g001204 may be different from *Pun4*.

**Table 4:** Conversion of physical positions of previously reported QTLs based on the Dempsey v1.0 reference genome

QTL	CM334 v.1.55			Dempsey v1.0			Co-localized gene	References
	Chromosome	Start (Mb)	End (Mb)	Chromosome	Start (Mb)	End (Mb)		
*TH-total1.1, TH-cap1.1*	1	2.6	7.9	1	3.5	− ^a^		Han et al. (2018)
*TH-cap1.2, TH-total1.2*	1	10.4	15.5	1	13.7	21.1		Han et al. (2018)
*TH-cap1.3*	1	15.5	20.1	1	21.1	25.5		Han et al. (2018)
*PD-dicap1.1, PD-cap1, PD-total1.1, TH-cap1.4*	1	39.7	60.7	1	57.5	81.5		Han et al. (2018)
*PD-dicap1.2, PD-dicap1.3, PD-total1.2*	1	201.7	222.0	1	222.4	254.3		Han et al. (2018)
*TH-cap1.5, TH-total1.3*	1	260.4	267.7	1	283.3	320.1		Han et al. (2018)
*TH-cap2.1*	2	120.6	124.2	2	116.5	120.7		Han et al. (2018)
*TH-total2, TH-cap2.2, PD-dicap2.1, PD-dicap2.2, PD-total2, PD-cap2, qdhc2.1, qdhc2.2*	2	124.8	132.3	2	126.6	167.6	*Pun1* (*CaAT3*)	Lee et al. (2016); Han et al. (2018)
*3.1*	3	0.0	1.7	3	5.3^b^	4.9		Yarnes et al. (2013)
*HJ-dhc3*	3	32.9	34.4	3	35.0	36.5	*pAMT*	Park et al. 2019
*TH-dicap3.1, TH-dicap3.2, TH-total3.1*	3	199.4	214.7	3	219.5	249.3		Han et al. (2018)
*TH-dicap3.3*	3	214.7	221.5	3	249.3	256.2		Han et al. (2018)
*TH-total3.2, PD-cap3*	3	225.1	237.8	3	257.3	271.1	*CSE* (CA03g24780)	Han et al. (2018)
*qcap3.1*	3	225.5	230.0	3	260.1	266.2	*CSE* (CA03g24780)	Lee et al. (2016)
*Shql3*	3	236.9	252.2	3	270.6	285.1		Kondo et al. (2023)
*TH-total3.3*	3	239.4	246.9	3	272.4	281.9	*3A2*, *4CL* (CA03g30500)	Han et al. (2018)
*cap3.1, total3.1*	3	247.5	247.9	3	283.4^b^	283.0	*3A2*, *4CL* (CA03g30500)	Ben-Chaim et al. (2006)
*4.2*	4	224.7	224.7	4	11.5	14.7		Yarnes et al. (2013)
*PD-total4.1, PD-dicap4*	4	16.3	21.4	4	18.0	29.9		Han et al. (2018)
*TH-cap4, PD-total4.2*	4	110.4	177.4	4	–^a^	191.1		Han et al. (2018)
*cap4.1, dhc4.1, total4.1, 4.13, 4.14, 4.15, 4.16*	4	202.8	221.9	4	224.2	243.6	*BCAT*	Ben-Chaim et al. (2006); Yarnes et al. (2013)
*cap4.2, dhc4.2, total4.2*	4	− ^a^	− ^a^	− ^a^	− ^a^	− ^a^		Ben-Chaim et al. (2006)
*5.4*	5	192.4	221.6	5	212.9	236.0		Yarnes et al. (2013)
*SJ-dhc6.1*	6	23.5	32.9	6	25.7	27.4		Park et al. (2019)
*HJ-tcp6.1, HJ-tcp6.2*	6	114.6	135.5	6	60.6	85.6		Park et al. (2019)
*qcap6.1*	6	121.0	175.8	6	150.3	188.2	*IPMS* (CA06g08090)	Lee et al. (2016)
*PD-cap6*	6	192.8	196.0	6	197.5	200.7		Han et al. (2018)
*SJ-dhc6.2, punv*	6	201.9	203.5	6	219.1^b^	212.0	*Pun4*, Capana06g001204	Park et al. (2019); Cao et al. (2022)
*HJ-tcp6.4 (2017)*	6	222.5	222.9	6	231.4	238.9		Park et al. (2019)
*HJ-tcp6.3*	6	223.8	225.7	6	231.7	235.2		Park et al. (2019)
*HJ-dhc6.1, HJ-tcp6.4 (2016)*	6	226.2	226.6	6	235.7	236.1		Park et al. (2019)
*HJ-dhc6.2*	6	226.9	227.1	6	239.8^b^	236.6		Park et al. (2019)
*SJ-cap6.1, SJ-dhc6.3, SJ-tcp6.1, SJ-cap6.2, SJ-tcp6.2*	6	232.1	237.4	6	241.8^b^	239.8		Park et al. (2019)
*6.8, TH-dicap6, TH-total6*	6	233.7	236.6	6	245.8	246.9	*FatA* (CA06g26640)	Yarnes et al. (2013); Han et al. (2018)
*cap7.1, dhc7.1, total7.1*	7	132.5	152.8	7	102.3	129.1		Ben-Chaim et al. (2006)
*7.3*	7	185.2	185.2	7	210.4	210.4	*Pun3* (*CaMYB31*)	Yarnes et al. (2013)
*cap, cap7.2, dhc7.2, total7.2*	7	202.6	203.5	7	231.2	232.1	*Pun3* (*CaMYB31*)	Blum et al. (2003); Ben-Chaim et al. (2006)
*Shql7*	7	218.7	231.0	7	248.4	265.1		Kondo et al. (2023)
*PD-dicap10.1, PD-total10.1*	10	3.6	8.8	10	3.7	9.2		Han et al. (2018)
*TH-total10, TH-dicap10, PD-cap10, PD-total10.2, PD-dicap10.2*	10	9.6	23.9	10	9.8	77.3		Han et al. (2018)
*10.2*	10	204.3	211.5	10	204.6	212.9		Yarnes et al. (2013)
*10.3*	10	217.9	218.2	10	221.5	221.8	*CaKR1* (CA10g18840)	Yarnes et al. (2013)
*HJ-tcp11*	11	29.3	29.4	11	28.1	28.2		Park et al. (2019)
*11.8*	11	245.8	246.6	11	267.1	268.0		Yarnes et al. (2013)
*PD-dicap12, PD-total12*	12	18.4	24.2	12	20.1	40.6		Han et al. (2018)

^a^ No clear position was detected based on BLASTn

^b^ The position of the flanking sequence is inverted in the genome context

### Genome context differences between Dempsey and Yuwolcho genome near the *Pun4* locus

Considering the potential failure in discovering variation due to contextual differences within the mapping region of Dempsey and Yuwolcho, we generated a Yuwolcho assembly. This resulted in a contig-level assembly and confirmed contextual differences between Yuwolcho and Dempsey through gene prediction and functional annotation (Fig. 5). And, the BSA-seq data was aligned to the Yuwolcho assembly to obtain the SNP list within the predicted gene sequences (Table S8). However, three SNPs (ptg000019l:9,538,501, ptg000019l:9,560,873, ptg000019l:9,610,609) that homogeneously preserved the EMS-induced sequence were not present within the coding sequence. It is expected that the gene found in the delimited region obtained through mapping will not contain the *Pun4* target gene, and that an external genetic factor will interact with the SNP found in the region.

### Candidate gene selection and further studies

By combining the results of all analyses presented in this study, we have selected five genes located in the proximity of the *Pun4* locus as high-confidence candidates, although none of them showed significant differences in their genic sequences between WT and the mutants. Enhancers can modulate the expression of genes located as far as 2–3 Mb away in metazoans, and this phenomenon has also been observed in crop plants, such as maize (Krivega and Dean 2012; Stam et al. 2002; Weber et al. 2016). Therefore, it is possible that one of the mutations may be within a long-distance cis-regulatory element region of the candidates, acting as an enhancer or silencer. Additionally, the SNPs in the mapping region may exist on the trans-regulatory genetic factors, including ncRNA or miRNA related to CBGs, which cannot be ruled out. Consequently, the five candidate genes could potentially serve as target genes for *Pun4*, acting as either enhancers or silencing factors. Furthermore, CBG homologs that are located on chromosome 6 and exhibit differential expression between WT and mutants may be considered as another set of potential genes that interact with the mutation(s) within the *Pun4* locus (Table S7).

### Supplementary Information

Below is the link to the electronic supplementary material.

Supplementary file1 (PPTX 1376 KB)

Supplementary file2 (XLSX 49 KB)

## Data Availability

The datasets generated and analyzed during the current study are available from the corresponding author on reasonable request.
