# Supplementary material for: Genetic characterization of a locus responsible for low pungency using EMS-induced mutants in Capsicum annuum L
Source: Theor Appl Genet. 2024 Apr 12;137(5):101. doi: 10.1007/s00122-024-04602-3 (PMC11014816; doi:10.1007/s00122-024-04602-3)
Supplement: Supplementary file 1 — Supplementary file1 (PPTX 1376 KB) [file 122_2024_4602_MOESM1_ESM.pptx]

## Slide 1
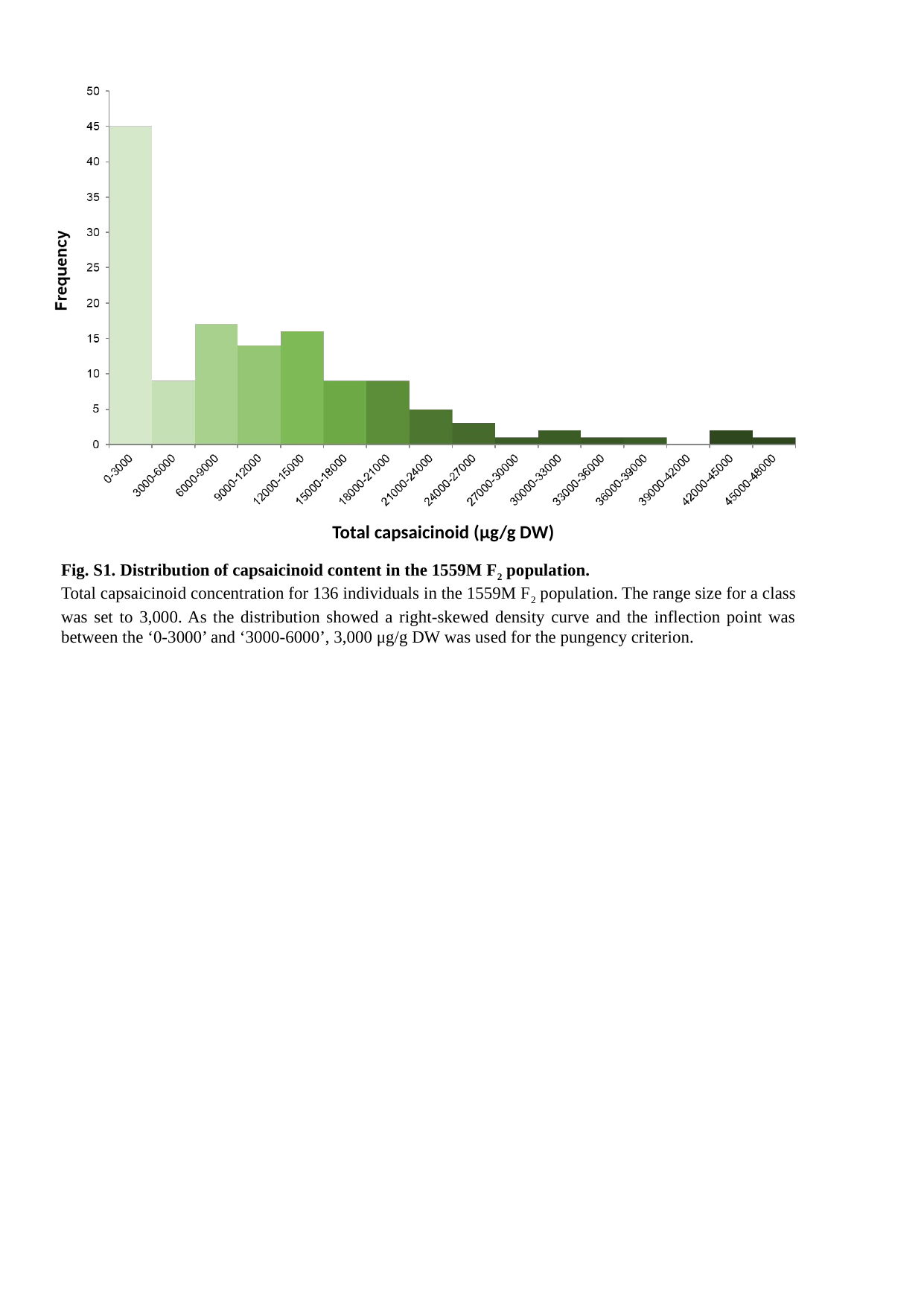

Total capsaicinoid (μg/g DW)
Fig. S1. Distribution of capsaicinoid content in the 1559M F2 population.
Total capsaicinoid concentration for 136 individuals in the 1559M F2 population. The range size for a class was set to 3,000. As the distribution showed a right-skewed density curve and the inflection point was between the ‘0-3000’ and ‘3000-6000’, 3,000 μg/g DW was used for the pungency criterion.

## Slide 2
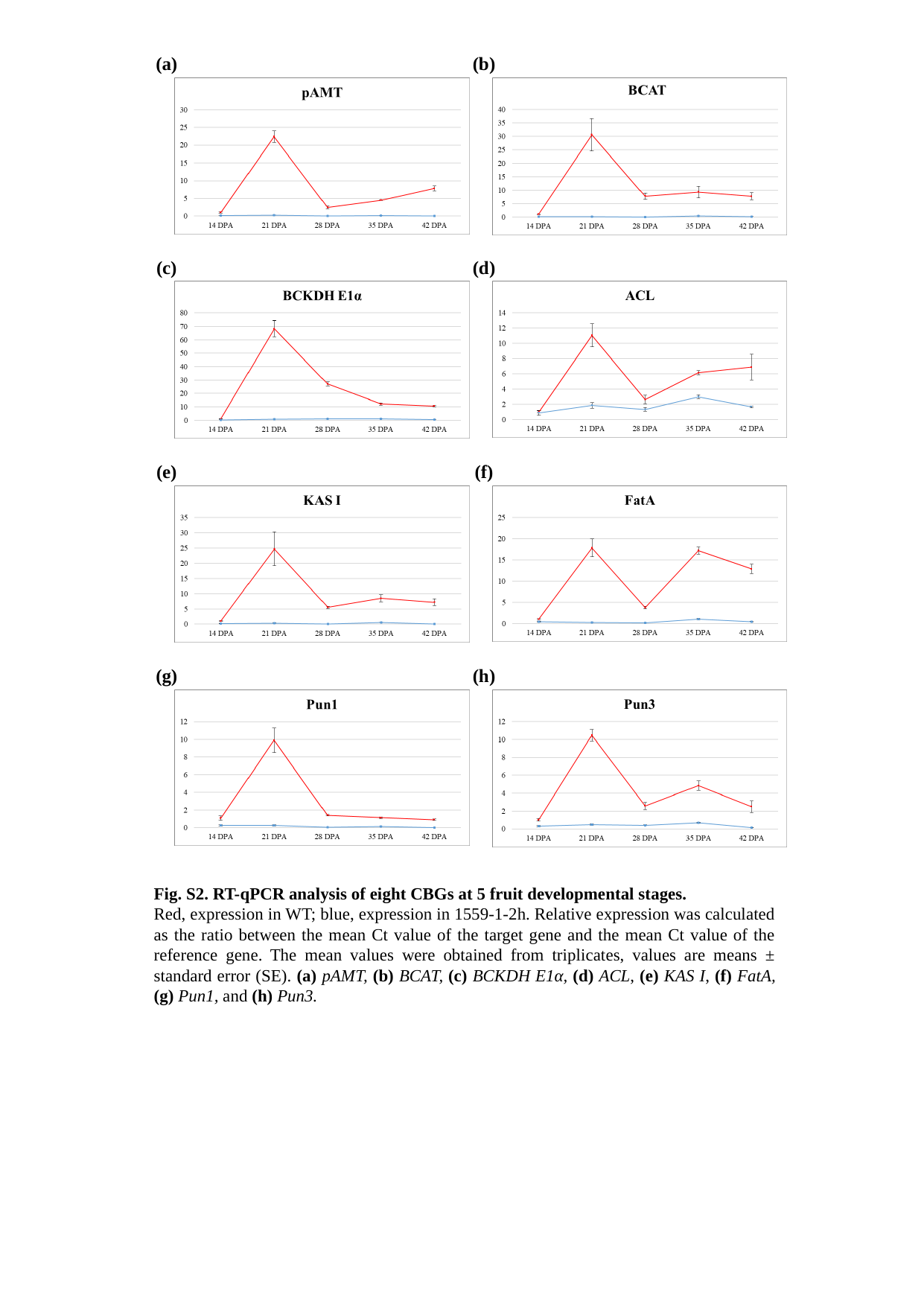

(a)
(b)
(c)
(d)
(e)
(f)
(g)
(h)
Fig. S2. RT-qPCR analysis of eight CBGs at 5 fruit developmental stages.
Red, expression in WT; blue, expression in 1559-1-2h. Relative expression was calculated as the ratio between the mean Ct value of the target gene and the mean Ct value of the reference gene. The mean values were obtained from triplicates, values are means ± standard error (SE). (a) pAMT, (b) BCAT, (c) BCKDH E1α, (d) ACL, (e) KAS I, (f) FatA, (g) Pun1, and (h) Pun3.

## Slide 3
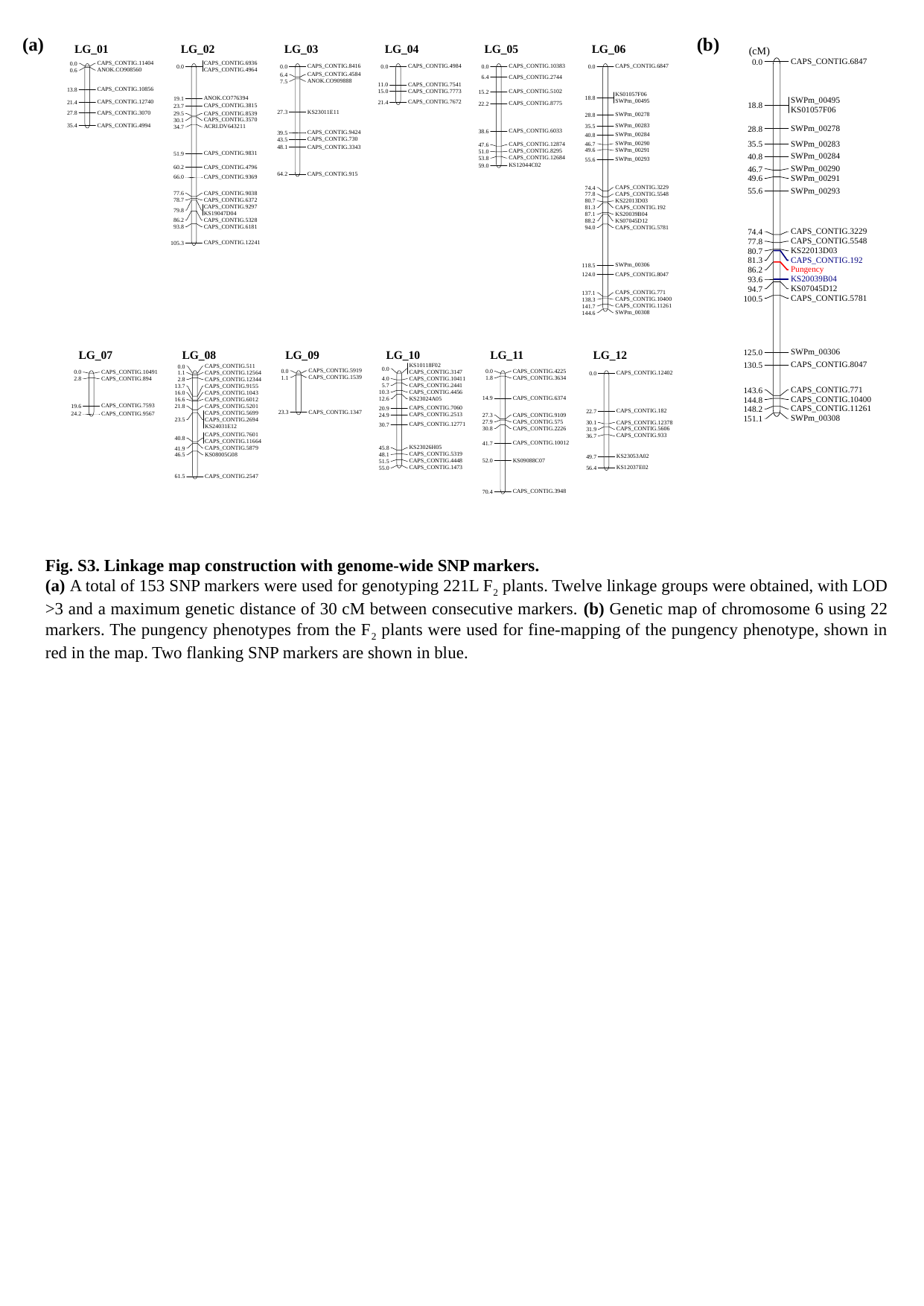

(a)
(b)
LG_01
LG_02
LG_03
LG_04
LG_05
LG_06
(cM)
LG_07
LG_08
LG_09
LG_10
LG_11
LG_12
Fig. S3. Linkage map construction with genome-wide SNP markers.
(a) A total of 153 SNP markers were used for genotyping 221L F2 plants. Twelve linkage groups were obtained, with LOD >3 and a maximum genetic distance of 30 cM between consecutive markers. (b) Genetic map of chromosome 6 using 22 markers. The pungency phenotypes from the F2 plants were used for fine-mapping of the pungency phenotype, shown in red in the map. Two flanking SNP markers are shown in blue.

## Slide 4
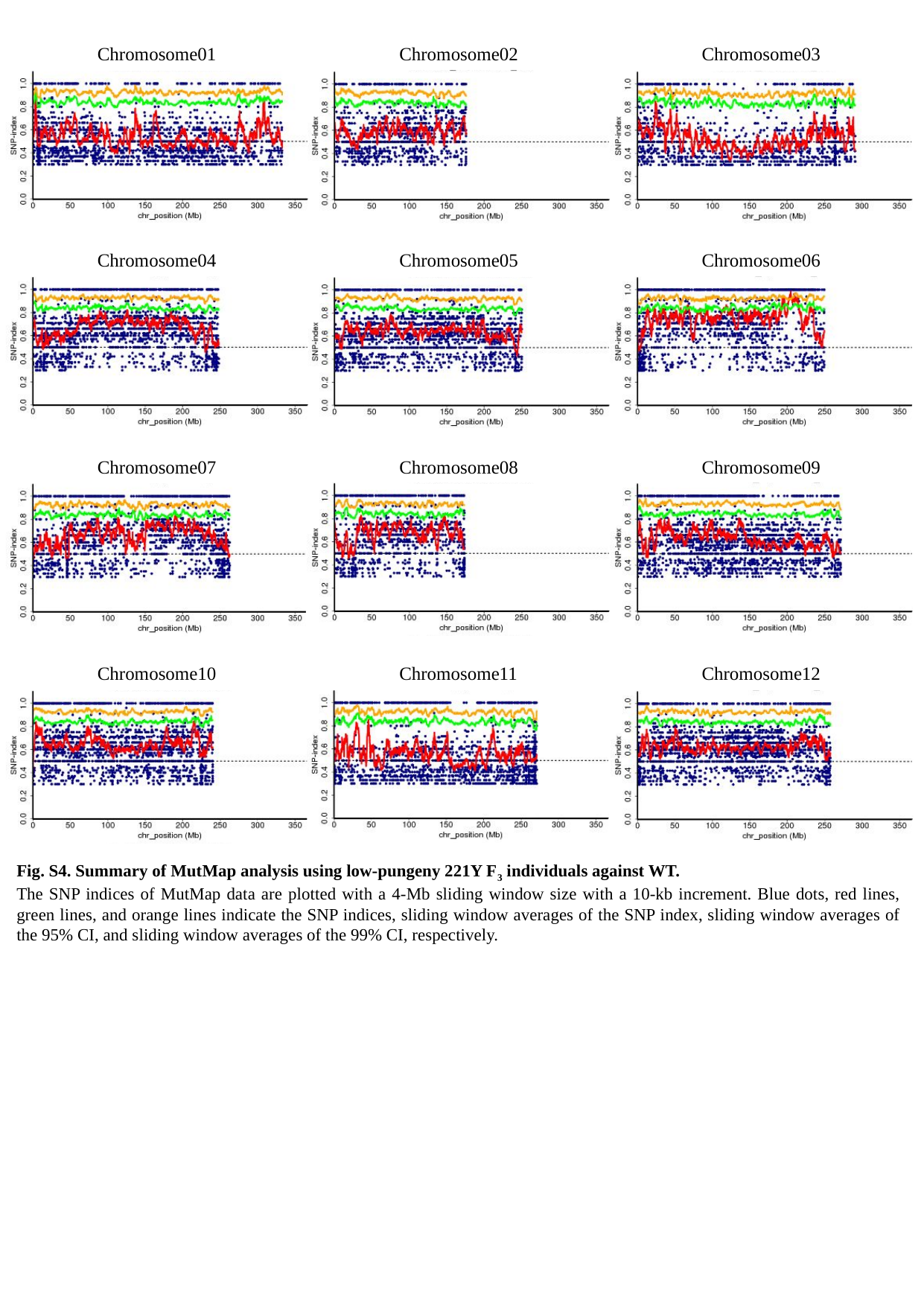

Chromosome01
Chromosome02
Chromosome03
Chromosome04
Chromosome05
Chromosome06
Chromosome07
Chromosome08
Chromosome09
Chromosome10
Chromosome11
Chromosome12
Fig. S4. Summary of MutMap analysis using low-pungeny 221Y F3 individuals against WT.
The SNP indices of MutMap data are plotted with a 4-Mb sliding window size with a 10-kb increment. Blue dots, red lines, green lines, and orange lines indicate the SNP indices, sliding window averages of the SNP index, sliding window averages of the 95% CI, and sliding window averages of the 99% CI, respectively.

## Slide 5
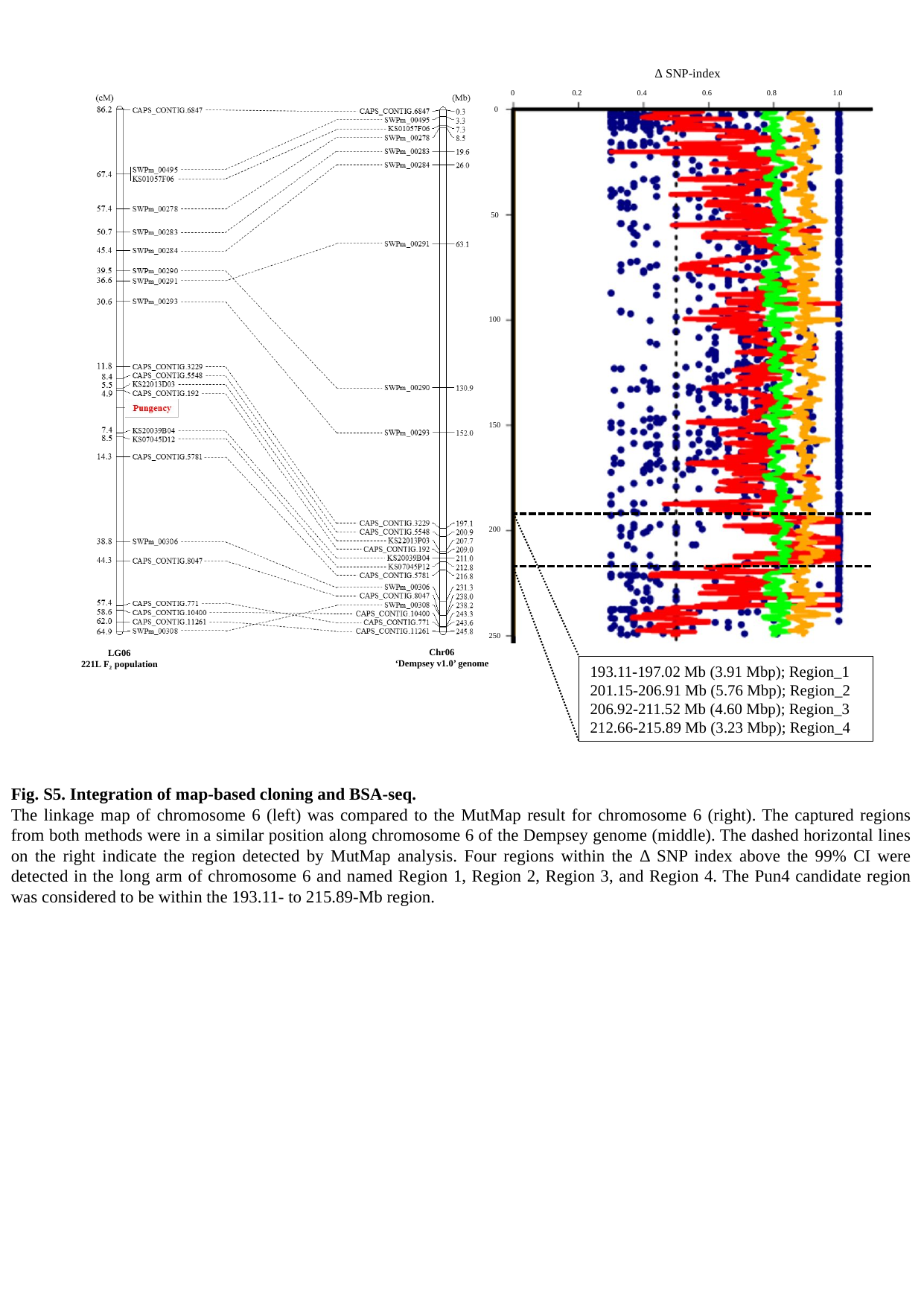

Δ SNP-index
0
0.2
0.4
0.6
0.8
1.0
0
50
100
150
200
250
LG06
221L F2 population
Chr06
‘Dempsey v1.0’ genome
193.11-197.02 Mb (3.91 Mbp); Region_1
201.15-206.91 Mb (5.76 Mbp); Region_2
206.92-211.52 Mb (4.60 Mbp); Region_3
212.66-215.89 Mb (3.23 Mbp); Region_4
Fig. S5. Integration of map-based cloning and BSA-seq.
The linkage map of chromosome 6 (left) was compared to the MutMap result for chromosome 6 (right). The captured regions from both methods were in a similar position along chromosome 6 of the Dempsey genome (middle). The dashed horizontal lines on the right indicate the region detected by MutMap analysis. Four regions within the Δ SNP index above the 99% CI were detected in the long arm of chromosome 6 and named Region 1, Region 2, Region 3, and Region 4. The Pun4 candidate region was considered to be within the 193.11- to 215.89-Mb region.

## Slide 6
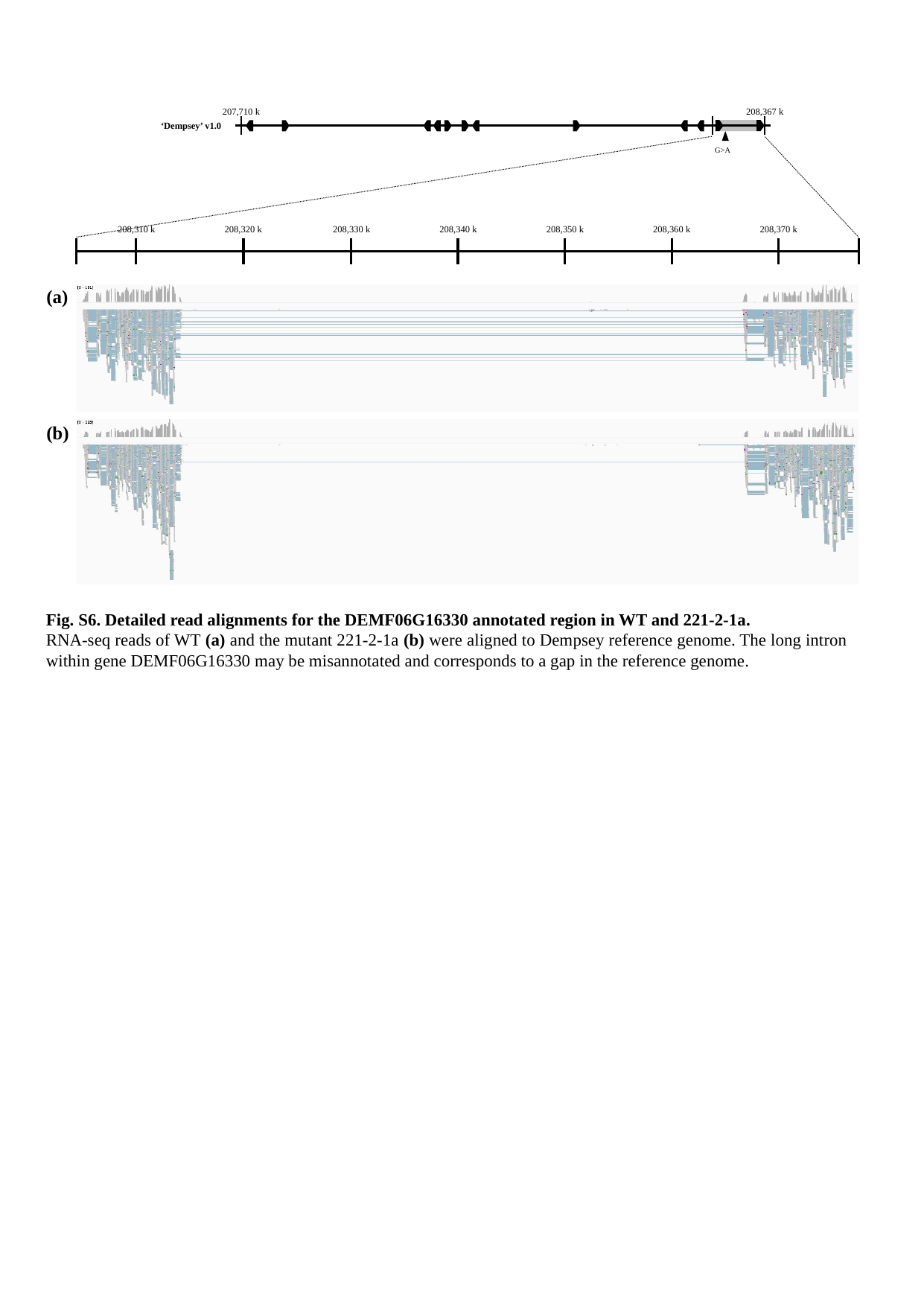

207,710 k
208,367 k
‘Dempsey’ v1.0
G>A
208,310 k
208,320 k
208,330 k
208,340 k
208,350 k
208,360 k
208,370 k
(a)
(b)
Fig. S6. Detailed read alignments for the DEMF06G16330 annotated region in WT and 221-2-1a.
RNA-seq reads of WT (a) and the mutant 221-2-1a (b) were aligned to Dempsey reference genome. The long intron within gene DEMF06G16330 may be misannotated and corresponds to a gap in the reference genome.

## Slide 7
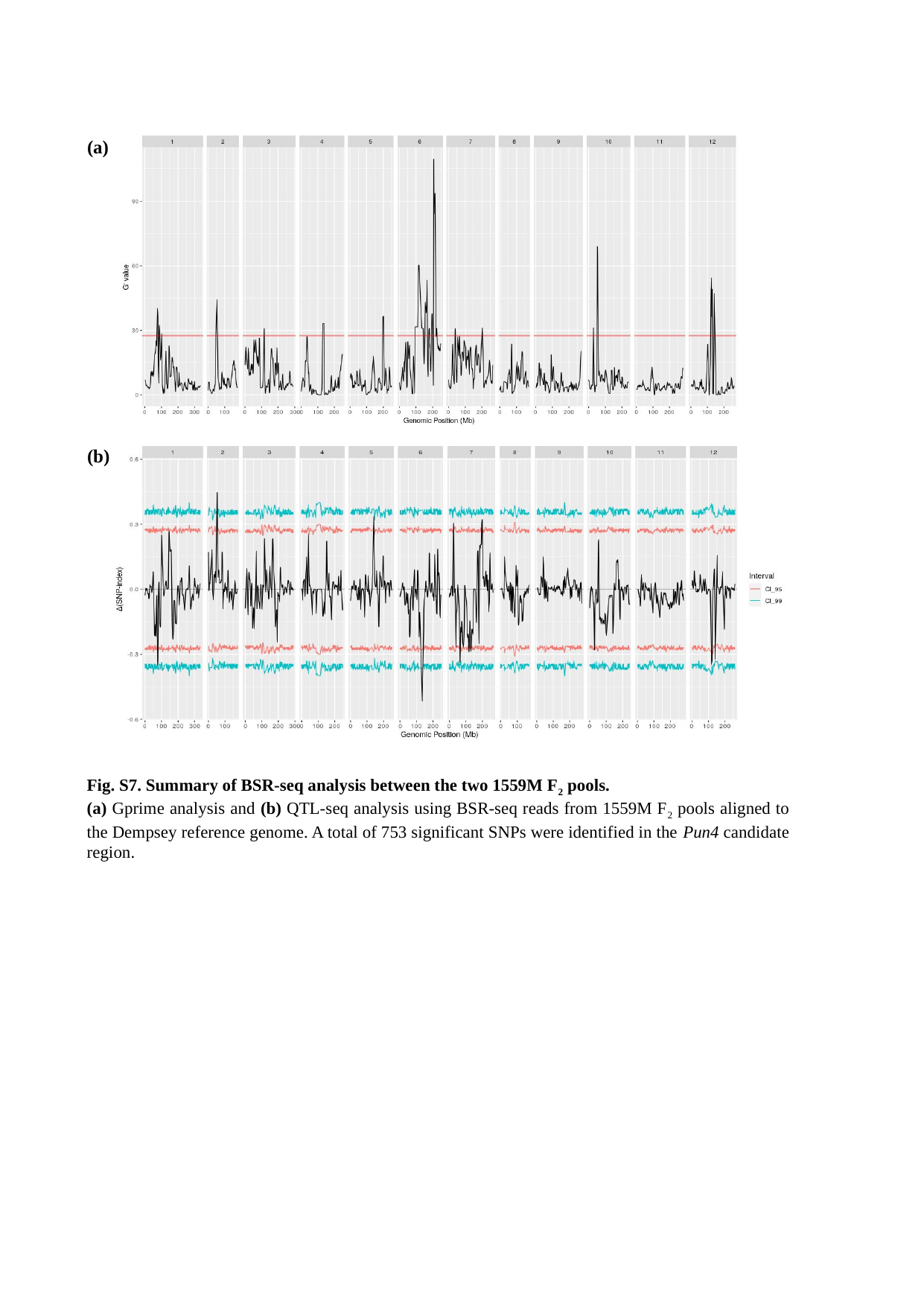

(a)
(b)
Fig. S7. Summary of BSR-seq analysis between the two 1559M F2 pools.
(a) Gprime analysis and (b) QTL-seq analysis using BSR-seq reads from 1559M F2 pools aligned to the Dempsey reference genome. A total of 753 significant SNPs were identified in the Pun4 candidate region.
